# Combined experimental and TD-DFT/DMOl^3^ investigations, optical properties, and photoluminescence behavior of a thiazolopyrimidine derivative

**DOI:** 10.1038/s41598-022-19840-y

**Published:** 2022-09-19

**Authors:** Amina A. Abozeed, Osama Younis, Ahmed F. Al-Hossainy, Nada Abd El-Mawla, Mostafa Sayed, Adel M. Kamal El-dean, Mahmoud S. Tolba

**Affiliations:** 1 Physics Department, Faculty of Science, Assiut University, Assiut, 71516 Egypt; 2 Chemistry Department, Faculty of Science, New Valley University, El-Kharga, 72511 Egypt; 3 Department of Chemistry, University of Science and Technology of China, Hefei, 230026 China; 4 Chemistry Department, Faculty of Science, Assiut University, Assiut, 71516 Egypt

**Keywords:** Particle physics, Materials chemistry

## Abstract

We present here the FT-IR, DFT computation, XRD, optical, and photophysical characterization of a heterocyclic compound with thienopyrimidine and pyran moieties. TD-DFT/DMOl^3^ and TD-DFT/CASTEP computations were used to study the geometry of isolated and dimer molecules and their optical behavior. The indirect (3.93 eV) and direct (3.29 eV) optical energy bandgaps, HOMO–LUMO energy gap (3.02 eV), and wavelength of maximum absorption (353 nm) were determined in the gas phase with M062X/6-31+G (d, p). A thin film of the studied molecule was studied using XRD, FT-IR, and UV–Vis spectroscopy. The average crystallite size was found as 74.95 nm. Also, the photoluminescence spectroscopy revealed that the compound exhibited different emission bands at the visible range with different intensities depending on the degree of molecular aggregation. For instance, solutions with different concentrations emitted blue, cyan, and green light. On the other hand, the solid-state material produced a dual emission with comparable intensities at λ_max_ = 455, 505, and 621 nm to cover the entire visible range and produce white emission from a single material with CIE coordinates of (0.34, 0.32) that are very similar to the ideal pure white light. Consequently, these findings could lead to the development of more attractive new luminous materials.

## Introduction

Purely organic light-emissive materials have potential biomedical and optoelectronic applications^1–3^. Multicolor emissive systems, particularly dual emitters, have many potential applications in data encryption^4^, sensor^5^, bioimaging^6^, anticounterfeiting^4^, and inexpensive efficient white organic light-emitting diodes (WOLEDs)^7^. Generally, the construction of worlds requires covering the entire visible light spectrum from 400 to 700 nm by the use of two (blue and yellow) or three (blue, green, and red) color emitters^8–10^. Complex device designs, time-consuming fabrication procedures, and high costs are usually required to accommodate several emissive materials^11^. Single-molecular systems emitting white light have various benefits over multi-fluorescent molecule systems: Single-molecular systems eliminate the drawbacks of degradation, color aging, and phase separation; they also result in increased reproducibility and stability, as well as simplified device fabrication^12–14^. Recently, we have developed several single-component light-emitting materials^15–19^. Nevertheless, designing pure organic molecules with a dual emission that is appropriate for useful WOLEDs needs a clear understanding of the dual emission mechanism. Owing to the complexity of engineering the dual photoluminescence combinations, studying the mechanism of dual emission is primarily essential and complicated as it requires taking into account both the molecule and its surroundings in solution, crystalline form, or thin layer^20^. This conformational isomerism, obtained by restricting covalent interactions at distinct dihedral angles, produces diverse spatial configurations of the atoms of the molecule^21^. Photophysical features of the conformers are particularly distinguishable because of alterations in electron interactions and distribution, which result in unique light emissions from each conformer^22,23^. Additionally, supramolecular structures and noncovalent interactions between molecules in some materials can affect the excited state and, as a result, the emission properties^24^. Thus, controlling these noncovalent interactions may enable tuning the relative intensities of the dual emission through the visible range to get white-light emission. Some luminescent heterocyclic compounds have been developed and showed a dependence of the emission characteristics on molecular interactions and aggregation^25–27^.

So, in this work, we introduce the synthesis, FT-IR, DFT computational study, XRD, optical, and photophysical characterization of a heterocyclic molecule with thienopyrimidine and pyran moieties. The studied compound gave white emission in the solid state from a single chromophore.

## Experimental tools and measurements

### Synthesis

**Figure 1:**
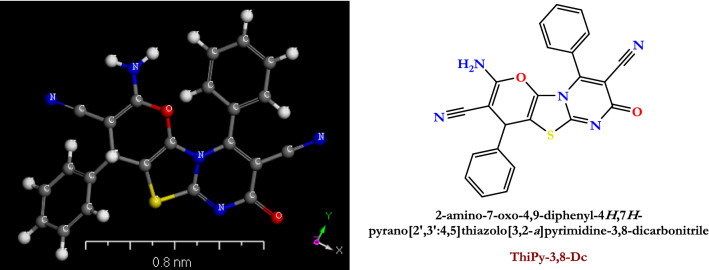
The chemical structure and 3D of the fabricated organic compound ThiPy-3,8-Dc.

The studied compound **[ThiPy-3,8-Dc]**, with the chemical structure and three dimensions shown in Fig. 1, was synthesized and purified according to the literature^28^.

### Preparation of the thin film [ThiPy-3,8-Dc]^TF^

Film preparation was performed using a spin coating system, and the deposition of pure **[ThiPy-3,8-Dc]**^**TF**^ thin film was carried out by dropping a 0.05 M dimethyl sulfoxide (DMSO) solution of **[ThiPy-3,8-Dc]** [C_23_H_13_N_5_O_2_S, New Valley University Lab, MW = 423.45] on glass substrates. Before the solution drop casting, ultrasonic cleaning with deionized water then methanol/acetone solutions were performed for 10 min on the glass substrates prior to drying with nitrogen. Glass substrates were rotated at 1000 rpm for 60 s after the coating solution was applied. After spin coating, drying at 150 °C for 10 min removed the organic residues and evaporated the solvents. The process was repeated five times, and the finished films were annealed for 2 h at 400 °C in an air-filled furnace.

### Computational study of the isolated molecule [ThiPy-3,8-Dc]^Iso^

It is well-known that time-dependent density functional theory (TDDFT) yields substantial errors for the excitation energies of charge-transfer (CT) excited states, when approximate standard exchange–correlation (xc) functionals are used, for example, B3LYP^29^. The *Materials Studio 7.0 program* on TDDFT/DMol^3^ was used to optimize the molecular structure and perform the frequency calculations for the crystal models and isolated molecules^30^. Without computing, the excited states, the frequency of the simulated IR dependence of a time-dependent electrical field perturbation excitation energies, and transition probabilities are calculated. The TDDFT algorithm’s performance is dependent on the approximation exchange and correlation functional (xc-functional) and the basis set. Numerous functionals have been proposed for TDDFT calculations. The well-known and broadly used Becke3–Lee–Yang–Parr hybrid functional (B3LYP) was favored in this study over the parameter-free Perdew–Burke–Enzerhof hybrid functional (abbreviated as PB0 or PBE1PBE). The PB0-functional was recommended by Jaquemin et al. in studies of classical dyes. In critical cases, however, both functionals were used in this study^31,32^. We employed the correlation consistent polarized valence basis sets with added diffuse functions, double and triple-zeta Pople-type basis sets (6-31+G*) with different amounts of polarization and diffuse functions, and polarization consistent basis sets with diffuse functions. Smaller basis sets are generally less suited. The standard basis set 6-31+G* was generally employed in the calculation of the optimum geometry and the spectral excitation and more extended basis set only if the results were unsatisfactory^33^. According to former studies the mean absolute error (MAE) of the calculated TDDFT excitation energies without consideration of the solvent effect amounts to about 0.21 eV^34^. Reflex simulates X-ray tools/TD-DFT, neutron, and electron powder diffraction patterns based on models of crystalline materials. Reflex Plus offers a complete package for the determination of crystal structures from medium- to high-quality powder diffraction data^35^.

### Characterization

Table 1 shows the instruments used to characterize the compound under investigation.

**Table 1:** List of analysis methods and equipment.

Analytical methods	Types and models
FT-IR	Perkin-Elmer FT-IR type 1650 spectrophotometer
XRD	A RIGAKUU Ultimo IV XRD / Cu *Kα* radiation ( *λ* = 1.5418 Å)
Optical measurements	Shimadzu UV-3600 UV–Vis NIR spectrophotometer
Luminescence	Hitachi F-7100 fluorescence spectrometer equipped with a detector photomultiplier R928F

## Results and discussions

### FT-IR Spectroscopy

**Figure 2:**
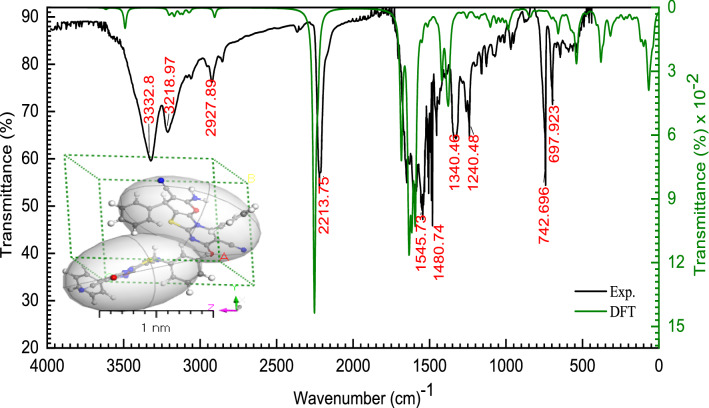
Experimental and theoretical FTIR; the inset is a 3D of an isolated molecule in the unit cell.

As shown in Fig. 2, the *DFT-Gaussian 09 W* vibration values are quite similar to the experimental data. Analyzing the theoretical infrared (IR) spectrum of **[ThiPy-3,8-Dc]**^**Iso**^ gave spectroscopic confirmation of its gaseous phase presence. Discrepancies between calculated and observed frequencies are shown in Fig. 2. While the calculations were done for the isolated molecules in the gaseous state, the measurements were performed for the thin film **[ThiPy-3,8-Dc]**^**TF**^. For **[ThiPy-3,8-Dc]**^**Iso**^ gaseous phase of an isolated molecule and **[ThiPy-3,8-Dc]**^**TF**^ thin film, the following equation has been used to define the direct correlation between the estimated $\left({Wn}_{Cal.}\right)$ and measured $\left({Wn}_{Exp.}\right)$ wavenumbers: ${Wn}_{Cal.}=0.015{Wn}_{Exp.}+5.28\mathrm{ with correlation coefficients }\left({R}^{2}= 0.98\right)$)^36,37^. The experimental and theoretical FTIR confirmed the presence of the bands characteristic of the functional groups such as stretching vibrations of NH_2_ at 3332.8 and 3218.9 cm^−1^, C–H at 2927.8 cm^−1^, C≡N at 2213.7 cm^−1^, C=O at 1649 cm^−1^, and aromatic C=C at 1545.7 cm^−1^.

### XRD structural analysis

**Figure 3:**
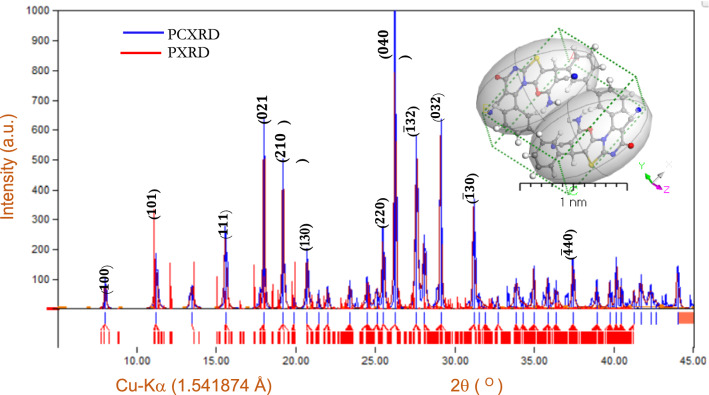
Comparison between the experimental (PXRD) and simulated XRD patterns (PCXRD), inset is a 3D triclinic lattice-type using *Polymorph computation method.*

The comparison between the powder XRD pattern of the thin film **[ThiPy-3,8-Dc]**^**TF**^ as experimental part (PXRD) and XRD pattern of *Polymorph computation method* of **[ThiPy-3,8-Dc]**^**Iso**^ as isolated molecule as simulated part (PCXRD) is displayed in Fig. 3. The intermolecular interference between **[ThiPy-3,8-Dc]**^**TF**^ chains may account for the strong peaks at 2θ at 19.3°, 26.3°, 27.7°, and 29.1°. Reflection of the triclinic symmetry in the space group corresponds to P-1^38^. According to database code amcsd 0001,845, d-spacing and miller index (*hkl*) corresponds to the actual value of 2θ^39^. At 2θ equals to 8.03°, 11.24°, 15.59°, 17.99°, 20.71°, 25.46°, 31.17°, and 37.40°, minor peaks were noticed. According to Al-Adiwish^40^, the base four peaks at 2θ = 19.19, 26.22, 27.56, and 29.09° correspond to 210, 040, $\overline{1 }$ 32 and 032 *hkl*, respectively, validate the crystal structure. Table 2 demonstrated the crystal parameters, *hkl*, d-spacing (d), as well as the full width at half-maximum (FWHM, ${\beta }_{hkl}$) of the crystalline structure. Figure 3 proves that the **[ThiPy-3,8-Dc]**^**TF**^ has a polycrystalline Å structure with a *Triclinic group* unit cell with the following characteristics a = 10.30 (4) Å, b = 20.1 (1) Å, c = 19.0 (2) Å, α = 900, β = 99.00 (4), γ = 900, and volume = 3800 (51) Å^3^^41^. Table 2 indicates that the average crystallite size (${D}_{Av}$) of **[ThiPy-3,8-Dc]**^**TF**^ is 74.95 nm. Because of the well-established relationship between the average crystallite size and the size distribution of semiconducting material, its properties have long been studied^42^. A wide dispersion spectrum in XRD patterns is dependent especially on the arrangements of atoms and crystallite size of particles in the unit cell^43^. At ${5}^{0}\le$ 2θ $\le {45}^{0}$, the FWHM (${\beta }_{hkl}$) and ${D}_{Av}$ were determined. The interplanar distance (d-spacing) between the greatest diffraction peaks and ${D}_{Av}$ was calculated using Bragg’ equation: ${D}_{Av}=0.9 \lambda /({\beta }_{hkl})cos\theta$ where (λ is the X-ray wavelength = 0.1541874 nm), θ is the matching 2θ, and the ${\beta }_{hkl}$ is FWHM (in radians)^44^ are recorded in Table 2.

**Table 2:** The computing results from the *Refine Version 3.0 Software Program (Kurt Barthelme’s and Bob Downs)* for **[ThiPy-3,8-Dc]**^**Iso**^ and **[ThiPy-3,8-Dc]**^**TF**^.

Symmetry	Experimental			Calculated		Difference		FWHM	D_av_^(b)^
	2θ	d	*hkl*	2θ	d	2θ	d		
Triclinic (P-1)	8.03	11.14	100	8.1081	11.027	0.0828	0.1149	0.1729	48.14
a = 11.64(2) nm	11.24	7.932	101	11.124	8.0169	−0.1175	−0.084	0.2407	34.66
b = 12.59(1) nm	15.59	5.712	111	15.64	5.6962	0.0444	0.0162	0.2252	37.22
c = 14.51 (2) nm	17.99	4.952	021	17.994	4.9519	0.0016	0.0004	0.1248	67.36
α = 102.79° (2)	19.19	4.643	210	19.179	4.6469	−0.0132	−0.003	0.1294	65.08
γ = 106.03°	20.71	4.304	130	20.733	4.3005	0.0207	0.0042	0.1494	56.50
β = 95.6° (2)	25.46	3.509	220	25.457	3.5091	−0.0005	−0.007	0.0819	103.94
V = 1960 (4)	26.22	3.408	040	26.211	3.4094	−0.008	−0.001	0.0564	151.17
Rmse^a)^ = 0.000181	27.56	3.244	−132	27.576	3.2431	0.0073	0.0008	0.2025	42.22
λ = 1.541838 Å	29.09	3.077	032	29.077	3.0785	−0.0107	−0.001	0.1046	82.01
Machine Error = − 0.097	31.17	2.876	−130	31.174	2.8754	0.0027	0.0002	0.0908	94.94
	37.42	2.407	−440	37.421	2.4072	0.0005	0.0034	0.0755	116.12
Average								22.74	74.95

(a) Root means square error.

XRD patterns of the polymorphs (PCXRD) were calculated utilizing the polymorph calculations approach in the *material studio software program (version 7.0)*. A $2\times 2\times 1$ matrix was used to calculate the integrals over the Brillouin zone, as shown in the inset of Fig. 3 (polymorph **[ThiPy-3,8-Dc]**^**Iso**^ as isolated molecule). There are slight changes in the intensities and position of certain peaks between the experimental and calculated XRD patterns; however, we will only concentrate on the major areas of similarity between them in this section^36^. Microstructural aspects of thin film samples may impact the experimental XRD pattern in addition to the features of instruments and data collecting methods. The slight changes include crystallite size, shape, and orientation distribution. The observed and calculated XRD patterns for both polymorphs match well when compared qualitatively, indicating that the synthesized material XRD patterns are correct^45^. Polymorph calculations of the XRD provide a fair indication of the atomic scale of the experimental findings at 2θ are 19.3°, 26.3°, 27.7°, and 29.1° as illustrated in Fig. 3.

### Geometry study of [ThiPy-3,8-Dc]^Iso^ and molecular electrostatic potential (MEP)

**Figure 4:**
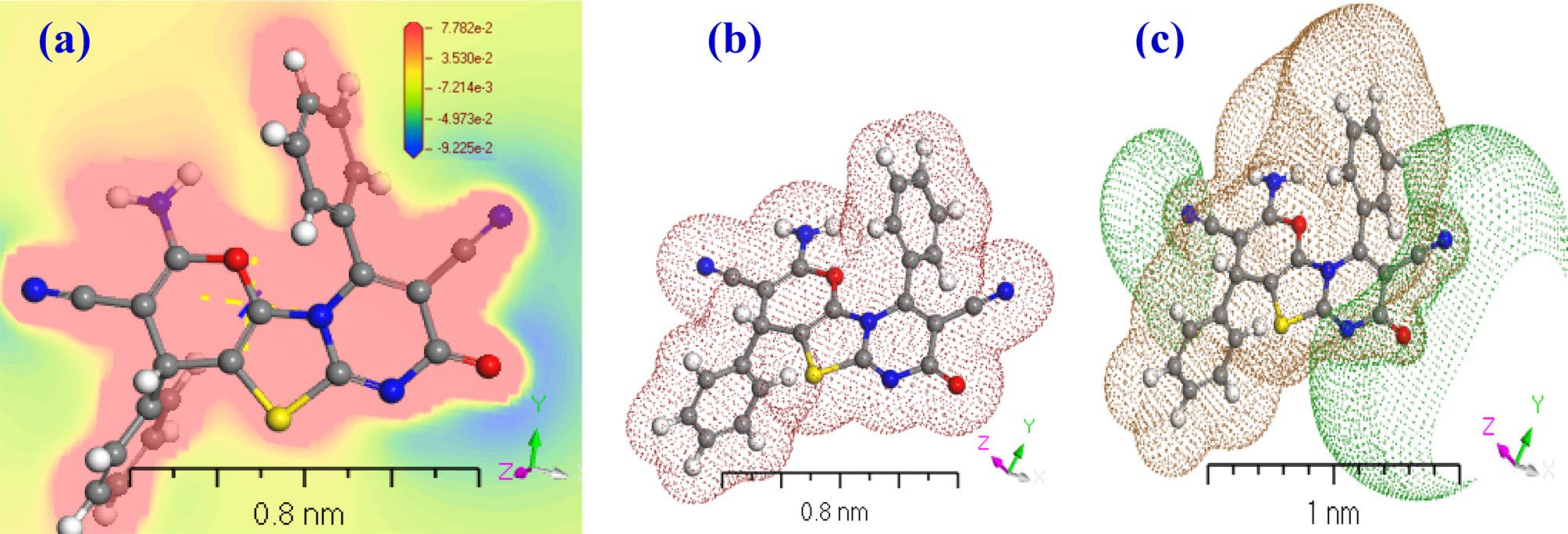
TD-DFT computation of (**a**) MEP of the **[ThiPy-3,8-Dc]**^**Iso**^, (**b**) Electron density and (**c**) Potentials of **[ThiPy-3,8-Dc]**^**Iso**^ using the DMOl^3^ method.

Using M062X/6-31+G(d,p) calculations, the HOMO and LUMO of the most stable conformer in the ground gaseous state were determined (Fig. 4). The energy difference between the frontier molecular orbitals (FMOs) determines the equilibrium state of the molecule, which is essential for calculating electrical conductivity and understanding electricity transit. Prior to applying modelling for **[ThiPy-3,8-Dc]**^**Iso**^ as an isolated molecule, the effect of numerous expressions on positive and negative surface ratios on electron levels were investigated. The difference between the average field and the negative and positive regions for a sample of over one thousand electron density molecules were examined. When MEPs are associated with 0.01–0.001 au, the data indicate an average 15% reduction in total. Until a specific number of nuclei is achieved, the positive surface density value remains stable whereas the number of negative sections reduces^46,47^. At 0.002 au, the percentage of positive area is approximately 68%; at 0.01 au, it exceeds 85%. In nanofluid pairs of fields, visual representations of the MEP Iso-surface value of − 15 kcal mol^−1^ can be used^48^, as shown in Fig. 4a. According to MEP topography, the calculated MEP_Vmin_ 3D minimum value closest to the lone pair area is 7.782 × 10^–2^ kcal/mol for [**ThiPy-3,8-Dc]**^**Iso**^. Using DMOl^3^/DFT designs, the [**ThiPy-3,8-Dc]**^**Iso**^ MEP_Vmax_ value is − 9.255 × 10^–2^ kcal/mol. As anticipated, the computed MEPV_max_ and MEPV_mim_ will take the electronic alternative into account. This uncommon relationship relies on [**[ThiPy-3,8-Dc]**^**Iso**^ energy. The principal characteristic of the negative MEPV_mim_ range is that it increases the electron density in a single pair of nitrogen atoms. To reduce the unfavorable lifetime of MEPVmim, an electron must be extracted from the cluster. MEPV_min_ concentrated on [**ThiPy-3,8-Dc]**^**Iso**^ electrical impact calculations, which may be more practical and clear than structures based on some factors like NH donation. Imagining [**ThiPy-3,8-Dc]**^**Iso**^ utilizes the organic compound matrix-donating strength. When [**ThiPy-3,8-Dc]**^**Iso**^ movement is not necessary, the energy exchange of [**ThiPy-3,8-Dc]**^**Iso**^ and MEPV_min_ are exactly equivalent^49^. The electron density in [**ThiPy-3,8-Dc]**^**Iso**^ is depicted in Fig. 4b. The negative electrostatic potential of the macro-cyclical plane is symmetrically distinct in all computations^50^, and the geometry of the mutually positive and mutually negative sections varies per base group. Figure 4c illustrates how the source range (DNP) is enlarged irrelevantly with the base folder (4.4), SCF lenience (0.0001), and maximum.

**Figure 5:**
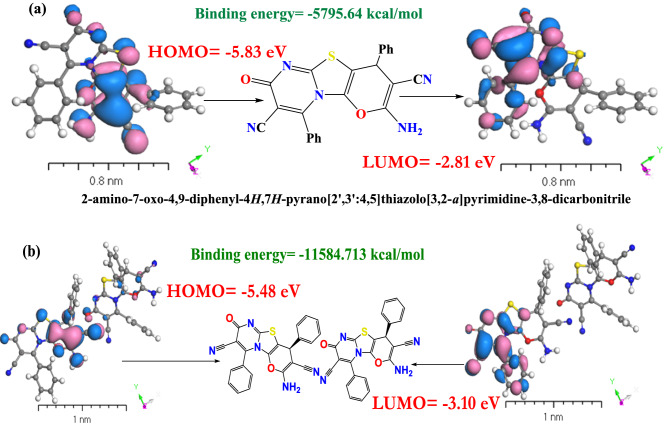
DFT computations using DMOl^3^ method for HOMO and LUMO calculations of (**a**) the isolated molecule and (**b**) the dimer.

The most stable molecular orbitals (HOMO and LUMO), in the ground gaseous state, were determined using M062X/6-31+G(d,p) computations, Fig. 5. The energy difference between the fragment molecular orbitals (FMOs) establishes the molecule equilibrium state, which is critical for estimating electrical conductivity and comprehending electricity transit. If the entropy values of isolated substances are negative, they are stable^51^. The observed FMOs can be used to determine the electrophilic sites of an aromatic molecule. When the number of dimer molecule bonds (DMB) increases and the bond length decreases, the Gutmannat variance approach is used in the DMB sites to increase the HOMO energy (${E}_{H}$)^52^. These properties were determined by examining the optimized energy gap (${E}_{g}^{Opt}$), and the molecular system reactivity and stability. The most crucial aspects in determining stability and responsiveness are the material softness and hardness^53,54^. Table 3 lists the computed electronegativity (χ) $= ({E}_{H}+{E}_{L})/2$ and ${E}_{g}^{Opt}$ which were used to demonstrate the charge transfer in the molecule. The HOMO level was commonly found on the C–NH_2_, –C–C–, $-\mathrm{C}\equiv \mathrm{N}$, and C–O–C atoms, which are primary targets for nucleophilic attack. From Fig. 5, we noticed that the HOMO energy of **[ThiPy-3,8-Dc]**^**Iso**^ in the gaseous state is − 5.83 eV which is a very low value, to indicate that **[ThiPy-3,8-Dc]** high excitation energies and stability. On the other hand, the lower LUMO energy value (− 2.81 eV) for **[ThiPy-3,8-Dc]**^**Iso**^ gaseous state is due to the high global softness (S). Soft molecules are referred to as reactive molecules since they can provide electrons to an acceptor. The electrophilicity (ω) of a device is critical because it contributes to the device’s energy stability by absorbing external electrical charges^38,55^. Electronegativity (χ = − μ), global hardness (η = (E_H_–E_L_)⁄2), chemical potential (μ = (E_H_ + E_L_)⁄2), softness (σ = 1⁄η), global electrophilicity index (ω = μ^2^⁄2η), global softness (S = 1⁄2η), and maximum amount of electronic charge (${\Delta N}_{max}$ = (− μ)⁄η) are presented in Table 3.

**Table 3:** The calculated $${E}_{H}$$, $${E}_{L}$$, energy gap ($${E}_{g}^{Opt})$$, global hardness (η), chemical potential (μ), electronegativity (χ), global softness (S), and global electrophilicity index (ω) for **[ThiPy-3,8-Dc]** as a dimer and a single molecule.

Molecule state	E_H_	E_L_	$\Delta {E}_{g}^{Opt.}$	χ (eV)	µ (eV)	η (eV)	S (eV)	ω (eV)	${\Delta N}_{max}$	σ(eV)^−1^
Single	−5.830	−2.810	3.020	4.320	−4.320	−1.510	−0.331	−6.180	−2.861	−0.662
Dimer	−5.480	−3.100	2.380	4.290	−4.290	−1.190	−0.420	−7.733	−3.605	−0.840

There were numerous conformers studied for the ground state geometry in quantum-chemical calculations and the conformer with the lowest energy was selected, which was validated by the harmonic vibrational frequency. The dimers binding energies were adjusted for the basis set superposition error using the counterpoise correction technique BSSE. The binding energies of **[ThiPy-3,8-Dc]** dimers and single molecules are + 5795.644 and + 11,584.712 kcal/mol, respectively^56^. Dimers binding energies ($\Delta {\mathrm{E}}_{\mathrm{b}}$) were assessed at the same level of theory using the following formula: $\Delta {\mathrm{E}}_{\mathrm{b}}={E}_{dimer}-2{E}_{monomer}=6.575 eV$.

**Figure 6:**
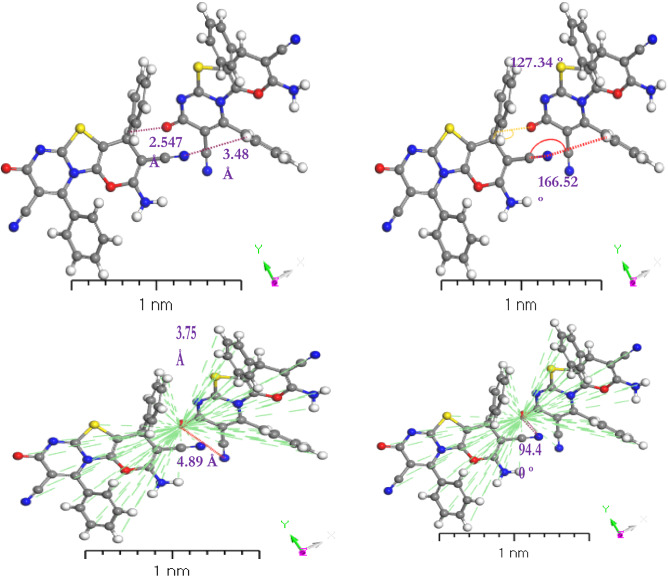
Stable structures of **[ThiPy-3,8-Dc]** dimers in the gas phase, calculated with B3LYP/6–31+G(d,p).

Recognizing the nature of noncovalent interactions between small nonpolar molecules is not only intriguing from a theoretical standpoint, but also essential for practical applications. Using the quantum chemistry computations and energy decomposition analyses, the interaction mechanism of **[ThiPy-3,8-Dc]** dimers is explored. Even though the monomers have zero dipole moment, it is demonstrated that their configuration choices are governed mostly by the electrostatic component and not the dispersion effect. These configuration choices can also be understood directly by analyzing the electrostatic potential map^57,58^. TDDFT/DMOl^3^ method was done on the studied molecules and their dimers to get a better understanding of the nature of intermolecular interactions^59^. Intermolecular hydrogen bonding interactions C–H…O, and C–H…N are shown in Fig. 6 for the examined molecule. The lengths of C–H…O and C–H…N hydrogen bonds are 2.547 Å and 3.48 Å, respectively. On the other hand, the dimer centroid lengths are 3.75 Å and 4.89 Å. The intermolecular distance between the two dimers is less than 3.50 Å, preventing the rotation of the rings of both molecules about the single bonds. While the dimer’s centroid length exceeds 3.50, the molecule rings rotate around the centroid point^60^. The dihedral angles of the single molecule and the dimer are 166.52° and 127.43°, respectively. According to the type of atom, the dihedral angle will shift from 166.52° to 127.43° when two isolated molecules are joined (as in the case of polymerization) via hydrogen and π bonding in the 2-amino-4-phenyl-4H-pyran-3-carbonitrile moiety.

### Optical properties

**Figure 7:**
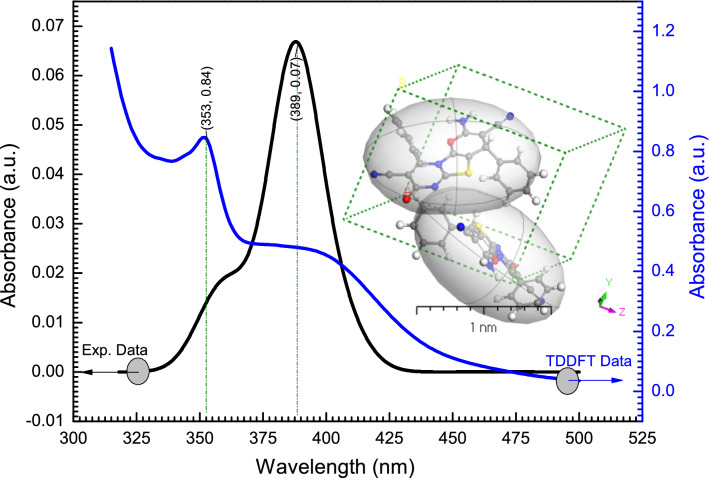
The absorbance spectra of **[ThiPy-3,8-Dc]**^**TF**^ thin film (Experimental part) and **[ThiPy-3,8-Dc]**^**Iso**^ as an isolated molecule in the gaseous state using TD-DFT/DMOl^3^ Technique. The inset figure is a 3D *Triclinic* lattice-type computed using the polymorph method.

Analyzing optical characteristics enables discussion of material energy gaps, band structure, and optical transitions. Figure 7 indicates the absorption spectra of the experimental **[ThiPy-3,8-Dc]**^**TF**^ thin film and the calculated **[ThiPy-3,8-Dc]**^**Iso**^ as a function of the photon wavelength (λ) in the range of the visible region. The experimental spectrum shows a shoulder at 358 nm and an absorption band at 389 nm (main peak), after 389 nm the absorption reduces significantly as the wavelength increases. This drop might be due to the decrease in the crystallinity of the films in this condition^61^. The TD-DFT analysis was used to examine the theoretical optical response of **[ThiPy-3,8-Dc]**^**Iso**^ as an isolated molecule at 600 > λ (nm) > 300. The TD-DFT/CASTEP findings were used to compare the absorption bands of **[ThiPy-3,8-Dc]**^**Iso**^ in DMSO as a solvent with the produced **[ThiPy-3,8-Dc]**^**TF**^ thin film with a thickness of 100 ± 5 nm developed at 298 K. The theoretical photoabsorption spectrum showed the main absorption band at 353 nm beside a shoulder at 389 nm. These peaks are attributed to the delocalized n–π* and π–π* transitions. By comparing the curve behavior and λ_max_ values obtained from the experimental approach and calculations, there is a good agreement between the experiment and the calculated results.

**Figure 8:**
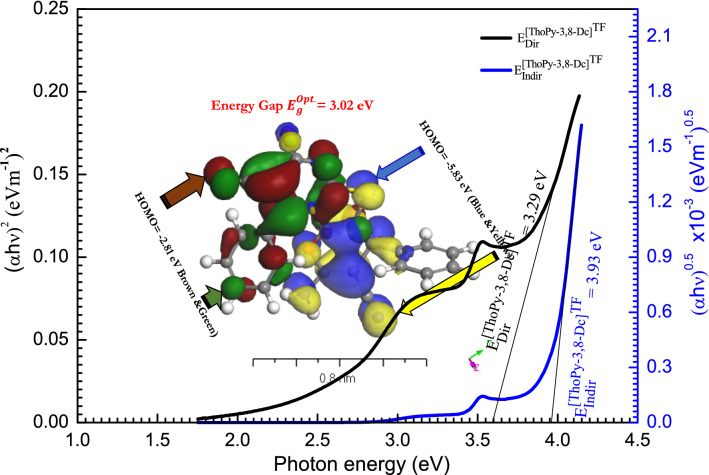
The relationship of photon energy with direct and indirect electronic transitions for **[ThiPy-3,8-Dc]**^**TF**^. The inset figure is a schematic diagram of theoretical calculations of the energy gap using TD-DFT/DMol^3^ method for the isolated molecule.

The indirect (${E}_{Indir}^{Opt.}$) and direct ${(E}_{Dir}^{Opt.})$ optical energy bandgaps were studied employing Tuac’s relation^62^, ${[(\alpha h\nu )}^{\psi }=\beta (h\nu -{E}_{g}^{Opt.})]$, where $(\beta )$ is a constant, $(h\nu )$ is the incident photon energy, and $(\psi =0.5 and 2)$ for the indirect and direct allowed transitions, respectively. ${(E}_{g}^{Opt.})$ is estimated from the straight portion of ${(\alpha h\nu )}^{2} a$ nd ${(\alpha h\nu )}^{0.5}$ versus $(h\nu$) plot at $\alpha =0$, as shown in Fig. 8. The estimated values of $({E}_{g}^{Opt.})$ are presented in Table 3. ${(E}_{Indir}^{Opt.})$ and ${(E}_{Dir}^{Opt.})$ of **[ThiPy-3,8-Dc]**^**TF**^ are 3.93 and 3.29 eV, respectively. The change in ${(E}_{g}^{Opt.})$ can be explained by the creation of the charge transfer between functional groups of ${(E}_{Dir}^{Opt.})$ and the amide groups of ${(E}_{indir}^{Opt.})$ which was reported in a previous work^63^. From the TD-DFT/DMOl^3^ calculations of HOMO and LUMO for **[ThiPy-3,8-Dc]**^**Iso**^ as an isolated molecule in a gaseous state (Fig. 8 inset), the value of ${E}_{g}^{Opt.}=3.02$ eV. suggesting that the TD-DFT functional reproduces the experimental values more accurately^64,65^.

**Figure 9:**
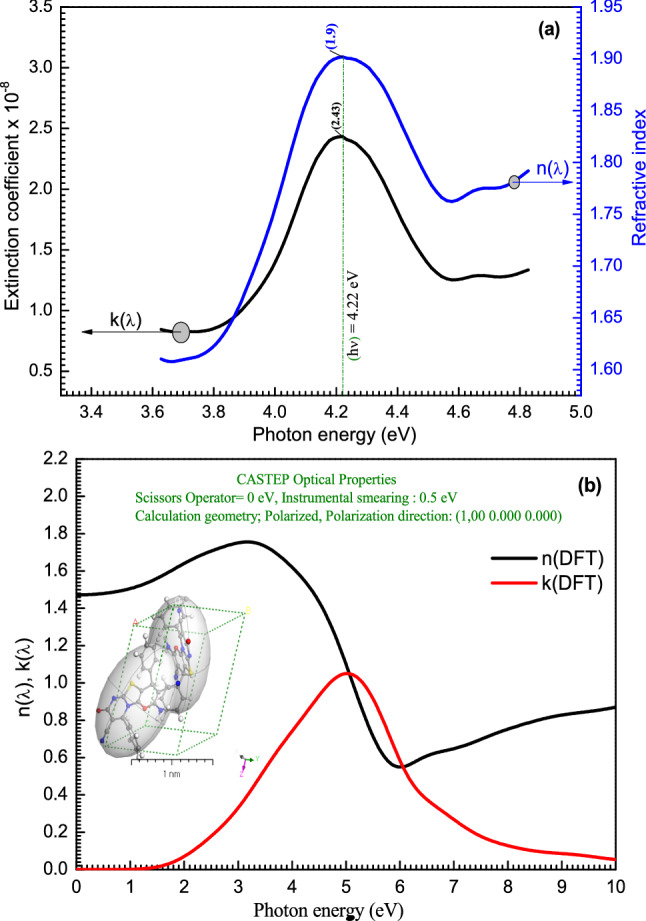
The extinction coefficient and refractive index of (**a**) **[ThiPy-3,8-Dc]**^**TF**^ and (**b**) simulated computations of the isolated molecule using CASTEP/DFT. The inset is a 3D triclinic lattice-type done using the polymorph computation method.

The refractive index is critical in the design of optical equipment and communication systems. Figure 9 demonstrates a comparison between the experimental extinction coefficient $(\lambda )$ and refractive index $n\left(\lambda \right)$ of **[ThiPy-3,8-Dc]**^**TF**^ and the simulated computations of **[ThiPy-3,8-Dc]**^**Iso**^ as an isolated molecule. When the experimental results are compared with the theoretical data (GGA-PW91 functional)^66^, a good agreement was found. The relationship between the extinction coefficient k (λ) of the films and the absorption (Abs.) spectra are as follows:$k=\alpha \lambda /4\pi$. The absorption coefficient $(\alpha )=Abs/d$ where d is the film thickness. Figure 9 shows the experiment and calculated $n(\lambda )$ and $k(\lambda )$ as a function of the photon energy of **[ThiPy-3,8-Dc]**. Both $n(\lambda )$ and $k(\lambda )$ have similar behavior, where both increase with increasing the photon energy until they reach the maximum values ($n(\lambda )=1.9$ and $k(\lambda )=2.43\times {10}^{-8}$) at 4.22 eV, then they start to decrease with increasing the photon energy. On the other hand, the value of k(λ) is less than the refractive index “n(λ)” with a ratio equal to $n(\lambda )/k\left(\lambda \right)=7.8\times {10}^{7}$. This result indicates that k(λ) is the key structural parameter controlling many canopy functions like radiation and water interception, radiation extinction, and water or gas exchange^67^. The CASTEP/DFT simulated technique was used to evaluate n(λ) and k(λ) and compared with the experimental values, a good similarity was obtained^68^.

**Figure 10:**
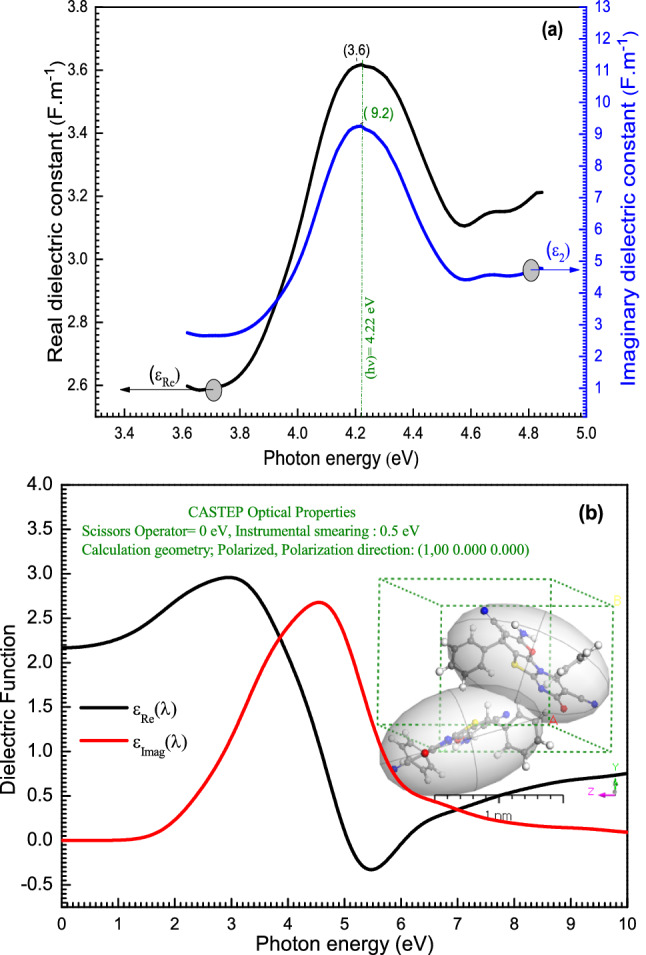
(**a**) The experimental ${\varepsilon }_{Re}$(λ) & ${\varepsilon }_{Imag}$(λ) for **[ThiPy-3,8-Dc]**^**TF**^. (**b**) Simulated ${\varepsilon }_{Re}$(λ) & ${\varepsilon }_{Imag}$(λ) for **[ThiPy-3,8-Dc]**^**Iso**^ as an isolated state using the CASTEP method.

The frequency dependence of the optical dielectric constant ($\varepsilon$) was supposed as an effective technique for elucidating electronic excitations in materials. The real and imaginary components of the complex permittivity characterize the dielectric features. $\varepsilon$ is defined as follows: $\varepsilon ={\varepsilon }_{1}-i{\varepsilon }_{2}$, where ${\varepsilon }_{1}$ is the real part (the dielectric constant) and ${\varepsilon }_{2}$ is the imaginary part (dielectric loss). The real and imaginary parts can be written in terms of $k(\lambda )$ and $n(\lambda )$ as follows: ${\varepsilon }_{1}={\varepsilon }_{real}={n(\lambda )}^{2}-{k(\lambda )}^{2}$, ${\varepsilon }_{2}={\varepsilon }_{imag}=2n(\lambda )k(\lambda )$^69^. It is seen, from Fig. 10a, that with increasing photon energy, the ${\varepsilon }_{Re }$ and ${\varepsilon }_{Imag}$ values increase and then decrease at the higher values of photon energy. The maximum values of ${\varepsilon }_{Re}$ and ${\varepsilon }_{Imag}$ are 3.61 and 9.2 × 10^–8^ ${(Fm}^{-1})$ at $h\nu = 4.22 eV$. The CASTEP/DFT technique is used to estimate ${\varepsilon }_{real}$ and ${\varepsilon }_{imag}$ values of **[ThiPy-3,8-Dc]**^**Iso**^ as an isolated state as a shown in Fig. 10b, the values of ${\varepsilon }_{Re}$(λ) and ${\varepsilon }_{Imag}$ (λ) in the isolated state are varied in the range of ≅ 0–3 ${(Fm}^{-1})$ at various photon energies (eV) ≅ 0–10. Reflecting that the experimental and calculated data illustrate similar behavior.

**Figure 11:**
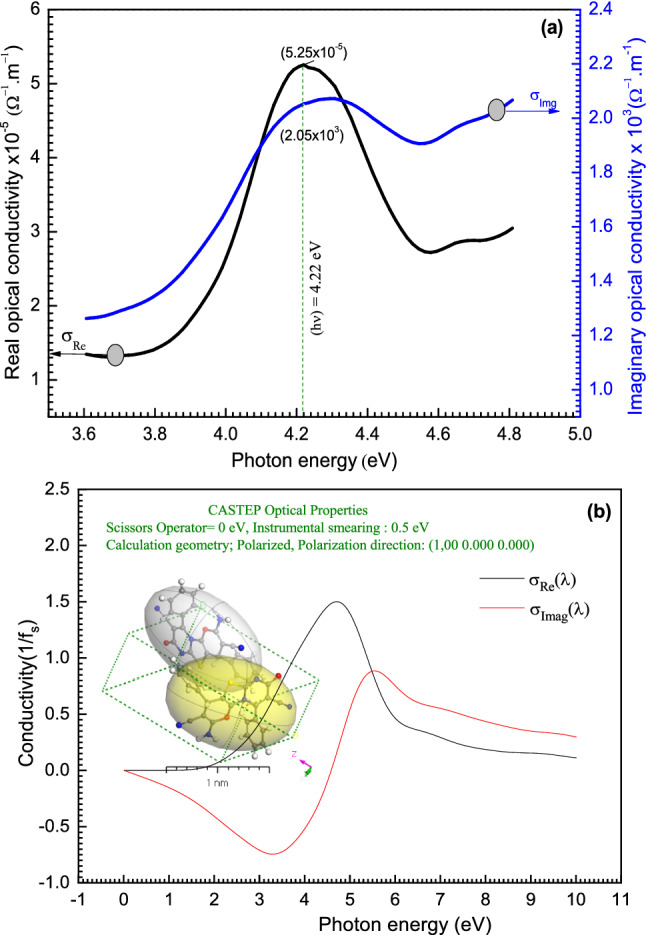
(**a**) The experimental σ_Re_(λ) and ${\sigma }_{Imag}$(λ) for **[ThiPy-3,8-Dc]**^**TF**^. (**b**) Simulated σ_Re_(λ) and ${\sigma }_{Imag}$(λ) for **[ThiPy-3,8-Dc]**^**Iso**^ as an isolated state using the CASTEP method.

Optical conductivity $\sigma (\lambda )$ explains the substance response to the electromagnetic wave. The optical conductivity real (${\sigma }_{Re}$) and imaginary (${\sigma }_{Imag}$) parts are calculated from^70^: ${\sigma }_{Re}\left(\omega \right)= \omega {\varepsilon }_{Imag}{\varepsilon }_{0}$ and ${\sigma }_{Imag}\left(\omega \right)= \omega {\varepsilon }_{Re}{\varepsilon }_{0}$ where the real part (${\sigma }_{Re}$) represents the in-phase current, while the imaginary part (${\sigma }_{Imag}$) represents the $\pi /2$ out-of-phase inductive current, ω is the angular frequency (ω = 2πν), ${\varepsilon }_{0}$ is the free space dielectric constant, ${\varepsilon }_{Re}$ is the real (normal) dielectric constant, and ${\varepsilon }_{Imag}$ is the imaginary (absorption associated with radiation by free carrier) parts of the dielectric constants. ${\varepsilon }_{Re}$ and ${\varepsilon }_{Imag}$ are given by ${\varepsilon }_{Re}={n}^{2}-{k}^{2}$ and ${\varepsilon }_{imag}=2nk$, respectively^71^. The dependence of ${\sigma }_{Re}$ and ${\sigma }_{Imag}$ on the incident photon energy ($h\nu$) is displayed in Fig. 10. The values of the imaginary part are larger than that of the real part of optical conductivity by a factor of ${\sigma }_{Imag}/{\sigma }_{Re}=4\times {10}^{7}$ (Fig. 11a). The values of ${\sigma }_{Re}$ and ${\sigma }_{Imag}$ are increased with the photon energy increasing till reaching the maximum value then start to decrease. At $h\nu =4.60$ eV, the conductivity is nearly constant and does like a plateau region. From the behavior of the simulated **[ThiPy-3,8-Dc]**^**Iso**^ as an isolated molecule in Fig. 11b, the CASTEP/DFT calculations were utilized to assess ${\sigma }_{Imag}$ and ${\sigma }_{Re}$ and compared to the experimental values of **[ThiPy-3,8-Dc]**^**TF**^, simulated values are close to those achieved by DFT with the CASTEP model.

### Photoluminescence behavior

**Figure 12:**
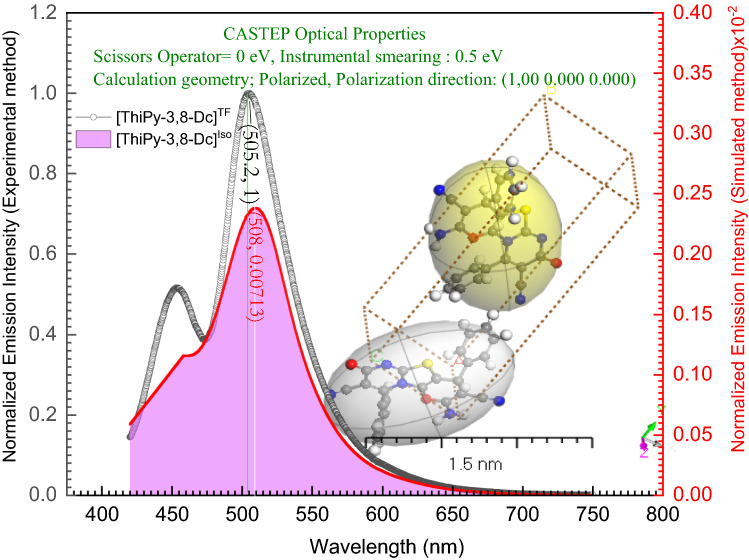
Combined between normalized emission spectra at *λ*_*max*_ = 400 nm of the solid at *λ*_*max*_ = 1 × 10^–5^ M (as Experimental) and simulated by using TD-DFT/CASTEP method.

**Figure 13:**
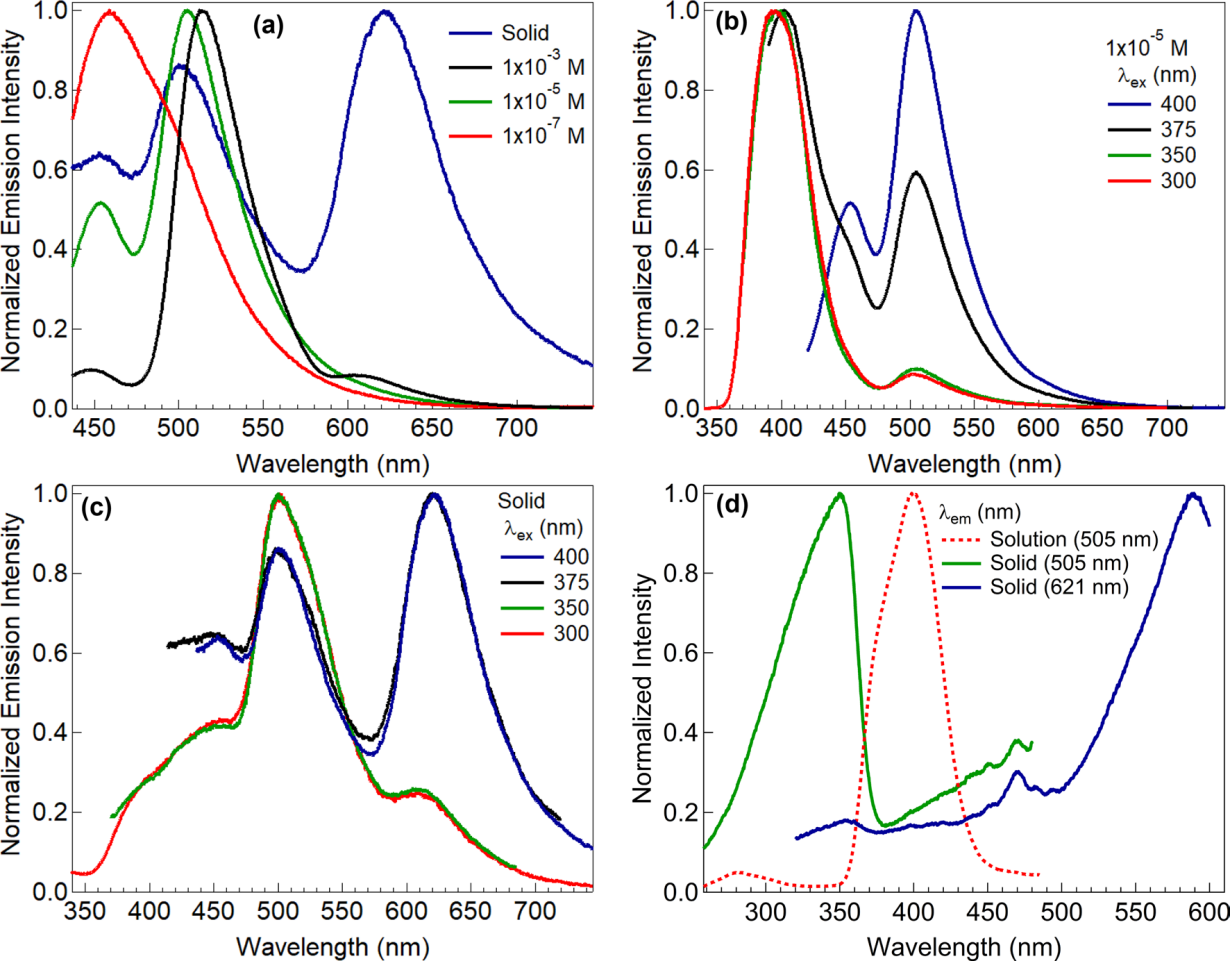
(**a**) Normalized emission spectra at *λ*_ex_ = 400 nm of the solid and different solution concentrations. (**b**) Normalized emission spectra at different *λ*_ex_ of the solution with a concentration of 1.0 × 10^–5^ mol L^–1^. (**c**) Normalized emission spectra at different *λ*_ex_ of the solid-state sample. (**d**) Excitation spectra of the solid and solution with a concentration of 1.0 × 10^–5^ mol L^–1^ at different *λ*_em_.

**Figure 14:**
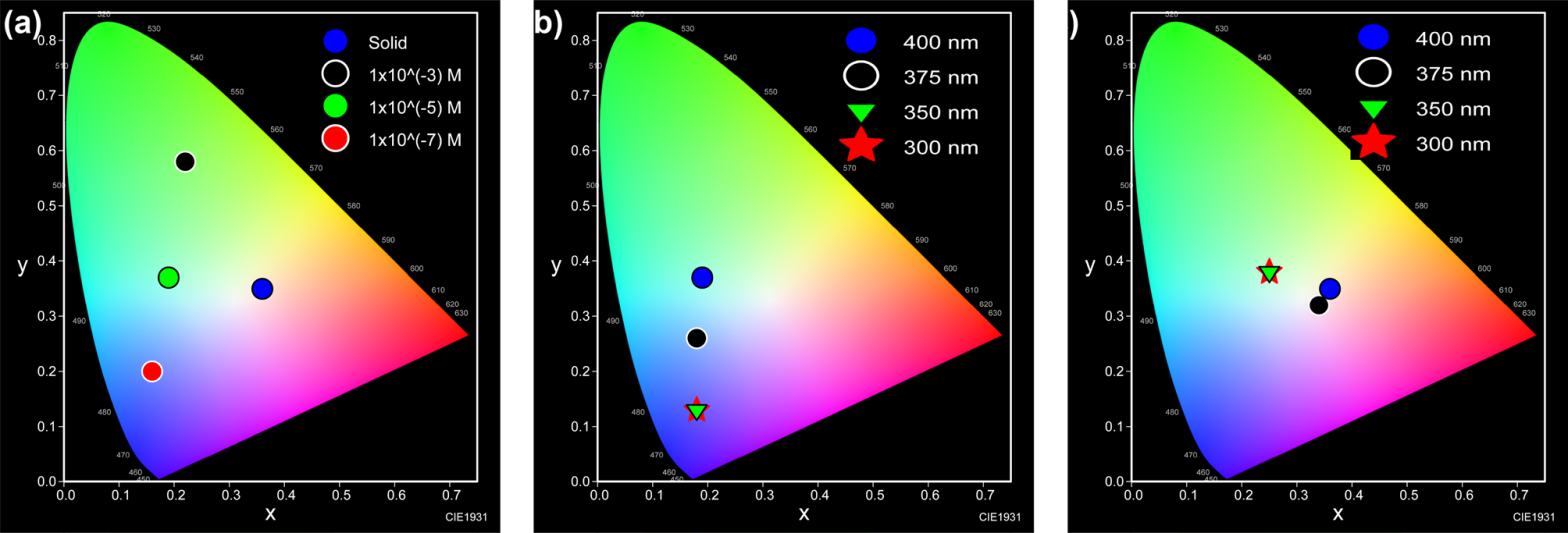
(**a**), (**b**), and (**c**) are the CIE chromaticity diagrams of the emission colors obtained from the spectra in Fig. 13a (the solid and different solution concentrations), Fig. 13b (different *λ*_ex_ of the solution), and Fig. 13c (different *λ*_ex_ of the solid), respectively.

The photophysical properties of the DMSO solution and powder of **[ThiPy-3,8-Dc]** were investigated at different solution concentrations and different excitation wavelengths. Also, the combination between the experimental emission spectra at λ_max_ = 400 nm of the solid and the simulated spectrum using TD-DFT/CASTEP method has been studied in Fig. 12. The maximum wavelength values are 505 nm and 508 nm for **[ThiPy-3,8-Dc]**^**exp**^ and **[ThiPy-3,8-Dc]**^**Iso**^, respectively. Thus, the value of ${\Delta \lambda }_{max}\hspace{0.17em}=\hspace{0.17em}$3 nm. From the behavior of the simulated **[ThiPy-3,8-Dc]**^**Iso**^ as an isolated molecule. The CASTEP/DFT calculations were utilized to assess normalized emission intensity and compared to the experimental values of **[ThiPy-3,8-Dc]**^**exp**^, simulated values are close to those achieved by TD-DFT with the CASTEP model. As seen in Fig. 13a, exciting **[ThiPy-3,8-Dc]** at 400 nm resulted in the emission at three main regions with λ_max_ = 455, 505, and 621 nm. The relative emission intensity of the high and low-energy emissions depends mainly on the degree of the molecular aggregation, where high diluted solutions (1.0 × 10^–7^ and 1.0 × 10^–5^ mol L^–1^) enhanced the high-energy emissions, while the low-energy peaks were predominated with more condensed phases such as the powder or the more concentrated solution (1.0 × 10^–3^ mol L^–1^). Thus, the high-energy emission at short wavelengths (455 nm) can be assigned to the single molecules intrachromophore π–π* transitions. Whereas. the low-energy bands at longer wavelengths (505 and 621 nm) would result from the strong π⋯π stacking of the aggregated molecules^72^. This red shift of the emission band is normal for organic-emissive molecules, where the created π–π interactions encourage the excimers creation^73–75^. Solutions of **[ThiPy-3,8-Dc]** emitted blue (1.0 × 10^–7^ mol L^–1^), cyan (1.0 × 10^–5^ mol L^–1^), and green light (1.0 × 10^–3^ mol L^–1^), CIE coordinates are listed in Table 4. These different emission colors may be due to the different molecular arrangements or packings. On the other hand, the solid-state material (powder) produced a dual emission with comparable intensities at 505 and 621 nm beside a shoulder at 455 nm to cover the entire visible range extending from 400 to 750 nm. CIE plot of this spectrum presented white-light emission from a single material with CIE coordinates of (0.34, 0.32) that are so close to the ideal coordinates of the pure white emission (0.33, 0.33). As concluded from the DFT simulations, a dimer of the studied molecule exhibited various kinds of molecular interactions with different molecular arrangements and tetrahedral angles from the isolated molecule. Thus, we speculate that at different molecular packings (solid or different solution concentrations), the noncovalent interactions (such as C–H···π, hydrogen bonding, and π···π stacking) stimulate various stacking arrangements with different emission behaviors^24^. In other words, the conformational heterogeneity relies mainly on the packing forces and molecular organization as well as the rigidity of the media. For example, the four states of the studied material (solid state and three different solution concentrations) showed various emission characteristics based on the ability to form specific intermolecular interactions and molecular arrangements which determined their photophysical properties. To explore the origin of the long-wavelength emissions, the excitation spectra were measured at different emission wavelengths (λ_em_ = 505 nm for the solution, 505 and 621 nm for the solid), Fig. 13d. Also, the photoluminescence spectra of **[ThiPy-3,8-Dc]** were measured for the solution and powder at different excitation wavelengths, λ_ex_ = 300, 350, 375, and 400 nm (see Fig. 13b,c. The obtained different excitation spectra imply different excited states. The emissions at different excitation wavelengths are not similar, thus proposing that these emissions should not have a common excitation pathway, to confirm the possibility of forming various fluorophores. Accordingly, controlling the degree of molecular aggregation can control the relative emission intensities of the three bands at 455, 505, and 621 nm to tune the luminescence color as seen from the Commission Internationale de l'Eclairage (CIE) chromaticity diagrams in Fig. 14.

**Table 4:** CIE coordinates of the emission colors for the spectra in Fig. 13.

Figure 13a			Figure 13b			Figure 13c		
State/conc. (mol L^–1^)	λ_ex_ (nm)	CIE (x, y)	State/conc. (mol L^–1^)	λ_ex_ (nm)	CIE (x, y)	State/conc. (mol L^–1^)	λ_ex_ (nm)	CIE (x, y)
Solid	400	0.36, 0.35	1.0 × 10^–5^	400	0.19, 0.37	Solid	400	0.36, 0.35
1.0 × 10^–3^	400	0.22, 0.58	1.0 × 10^–5^	375	0.18, 0.26	Solid	375	0.34, 0.32
1.0 × 10^–5^	400	0.19, 0.37	1.0 × 10^–5^	350	0.18, 0.13	Solid	350	0.25, 0.38
1.0 × 10^–7^	400	0.16, 0.20	1.0 × 10^–5^	300	0.18, 0.13	Solid	300	0.25, 0.38

## Conclusion

**[ThiPy-3,8-Dc]** emitted at different wavelengths of the visible range with relative emission intensities depending on the degree of molecular aggregation. The high-energy emission was assigned to the single molecules interchromophore π–π* transitions. Whereas the low-energy bands would result from the π⋯π stacking of the aggregated molecules. As concluded from the DFT simulations and emission spectra at different excitation wavelengths, the dimer of the studied molecule exhibited various molecular interactions and arrangements. Thus, we suppose that at different molecular packings, the noncovalent interactions (such as C–H···π, hydrogen bonding, and π···π stacking) promote various stacking arrangements with different emission behaviors. In other words, the conformational heterogeneity relies mainly on the packing forces and molecular organization as well as the rigidity of the media. For example, solutions of **[ThiPy-3,8-Dc]** emitted blue, cyan, and green light. On the other hand, the powder material produced a dual emission with comparable intensities that covered the entire visible range. CIE plot of this spectrum presented white-light emission from a single material with CIE coordinates of (0.34, 0.32) that are similar to the ideal coordinates of the pure white emission (0.33, 0.33). The four states of the studied material (solid and three different solution concentrations) showed various emission characteristics based on the ability to form specific intermolecular interactions and molecular arrangements.

## Data Availability

All data generated or analyzed during this study are included in this published article.
